# Gait dysfunction as an early marker of phenoconversion in REM sleep behavior disorder

**DOI:** 10.1038/s41598-026-37925-w

**Published:** 2026-02-09

**Authors:** Wiebke Hermann, Aleyna Sankutlu, Lydia Nabers, Tony Sehr, Katrin Trentzsch, Markus Donix, Heinz Reichmann, Moritz D. Brandt, Alexander Storch, Tjalf Ziemssen

**Affiliations:** 1 Department of Neurology, University Medical Center Rostock, University of Rostock, Schillingallee 35, 18147 Rostock, Germany; 2 Department of Neurology, University Hospital Carl Gustav Carus, Technische Universität Dresden, 01307 Dresden, Germany; 3 German Center for Neurodegenerative Diseases (DZNE), Research Site Rostock/Greifswald, 18147 Rostock, Germany; 4 Department of Psychiatry, University Hospital Carl Gustav Carus, Technische Universität Dresden, 01307 Dresden, Germany; 5 Department of Neurology, Elblandklinikum Meißen, 01662 Meißen, Germany

**Keywords:** REM sleep behaviour disorder, Progression, Α-synucleinopathies, Parkinson’s disease, Gait dysfunction, Diseases, Neurology, Neuroscience

## Abstract

Clinical isolated REM Sleep Behavior Disorder (iRBD) is a prodromal stage of α-synucleinopathies such as Parkinson’s disease (PD). Longitudinal changes of gait as a marker of phenoconversion in iRBD have not been systematically evaluated yet. Therefore, twenty-one iRBD patients, nineteen matched healthy controls and fourteen PD patients (H&Y stage I - III) were assessed at baseline using a pressure-sensor walkway (GAITRite). While normalized gait velocity and walking endurance were significantly decreased in iRBD patients compared to controls, gait rhythm parameters such as single support further discriminated PD from iRBD and controls. IRBD patients at imminent risk of phenoconversion within the next 3.7 ± 0.6 years were distinguishable from non-converters by gait rhythm parameters such as single support. Since gait evaluation of our iRBD cohort showed promising results in predicting imminent phenoconversion, future multicenter trials should include gait analysis as an objective assessment of motor function to confirm our results.

**Supplementary Information:**

The online version contains supplementary material available at 10.1038/s41598-026-37925-w.

## Introduction

Clinically isolated REM Sleep Behavior Disorder (iRBD), which is characterized by dream-enactment behavior during rapid eye movement (REM) sleep, has been recognized as the prodromal stage of α-synucleinopathies such as Parkinson’s disease (PD), Dementia with Lewy Bodies (DLB) and Multiple System Atrophy (MSA)^1–4^ with a conversion rate of 66% after 7.5years, around 76% at ten years, and 91% at 14 years^2,5^. Over years and decades a progressive worsening of clinical symptoms including cognitive, non-motor and motor abnormalities including gait disturbances to a varying degree is observed in all of these α-synucleinopathies. Disease-modifying therapies might be particularly effective in early disease stages^1,6^, thus leaving iRBD one of the most promising groups in future neuroprotective trials due to its high risk of phenoconversion^1,7^. Although various markers such as DATSCAN imaging have been proposed to identify iRBD patients at risk of imminent conversion, easily accessible and cost effective (bio-) markers to monitor progression over time and predict outcome need to be established^8^.

Gait abnormalities in the elderly are associated with dementia, mortality and other negative health outcomes^9^. Hereby, gait velocity showed an association with negative health outcome in general, while spatio-temporal gait parameters such as single and double support seem to reflect functional performance^9–12^. Furthermore, gait disturbances are one of the hallmark features of PD with subtle abnormalities in the early stages^13–15^ and progressive worsening over time^16^. While reduced amplitude of arm swing^17^, step length and gait velocity as well as increased double support time seem to occur relatively early^14,18–21^, other features such as gait initiation problems, freezing of gait and impaired postural control often occur later in the disease course^14,16^.

Although motor abnormalities are known to strongly predict phenoconversion^2^, the characteristics of gait abnormalities in iRBD as well as their potential to predict progressive neurodegeneration have not been ultimately determined, since longitudinal trials in these patients often focused on clinical instead of objective, rater-independent measures. A few small cross-sectional trials showed reduced gait velocity and differences in gait asymmetry parameters in iRBD patients compared to controls at regular walking speed, while more pronounced abnormalities in dual-task walking have been detected^22^. To our knowledge, only one recent small single-center trial evaluated clinical outcome based on gait parameters in iRBD patients longitudinally. Thus, the aim of our study was to determine the value of objective gait measurements to predict progression and phenoconversion in iRBD patients.

## Methods

### Study design, ethics and participants

This study comparing three groups of controls, iRBD and PD participants cross-sectionally as well as including longitudinal clinical data on iRBD was performed in accordance to the ethical principles of the Declaration of Helsinki (ICH). The study protocol and procedures were in accordance to the guidelines and regulations for human studies and were approved by the local Institutional Review Board (ethics committee of the Technische Universität Dresden, Dresden, Germany (EK-242072013, BO-EK-320062021)). Written informed consent was obtained from all study participants prior to study inclusion.

IRBD patients were recruited from inpatients of the Interdisciplinary Sleep Center of the Technische Universität Dresden, Germany, while PD patients were included as in- and outpatients at the Movement Disorder Center of the Technische Universität Dresden, Germany. Controls were recruited from patients referred to the Sleep Center for insomnia (*n* = 3), control of continuous positive airway pressure therapy for sleep apnea (CPAP, *n* = 5) or diagnosis of sleep apnea (*n* = 1). Furthermore, ten healthy volunteers were included, of which five were initially part of a study conducted by the University Memory Clinic^23^, while the other five healthy controls were recruited as healthy controls via the sleep laboratory or spouses of the iRBD patients. Out of over 106 participants screened for participation, we included 21 iRBD patients as well as 14 sex- and age matched PD patients and 19 controls into the analysis, while the others met exclusion criteria, which consisted of any condition possibly interfering with gait assessment such as diagnosis or clinical signs of either polyneuropathy, orthopedic causes such as joint replacement therapies or lumbar or cervical spinal stenosis.

PD diagnosis was based on UK Brain Bank criteria^24^. Diagnosis of iRBD was based on a positive history of dream-enactment behavior according to AASM criteria confirmed by polysomnography (PSG) using the SINBAR-protocol^25,26^, while also all controls underwent PSG to rule out REM sleep without atonia (RSWA). Furthermore, all participants underwent a detailed clinical and neurological assessment including the Unified Parkinson’s Disease Rating Scale (UPDRS) and Montreal Cognitive Assessment (MoCA) at baseline thus excluding controls and iRBD patients with MoCA < 26 and/or UPDRS III scores higher than 3 points in the control cohort and higher than 4 points in the iRBD cohort. Demographic details and medical history were obtained in all subjects including several questionnaires to screen for autonomic symptoms (Parkinson’s disease non-motor symptoms quest (PD-NMS)), depression (Beck’s Depression Inventory (BDI-II)), sleep (REM Sleep Behavior Disorder Screening Questionnaire (RBDSQ), Pittsburgh Sleep Quality Index (PSQI), Parkinson’s Disease Sleep Scale 2 (PDSS-2)) and daytime sleepiness (Epworth Sleepiness Scale (ESS)). Levodopa equivalent dose was calculated according to previously published reference values^27^.

Baseline gait assessment was performed between 2016 and 2018, while clinical follow-up visits in iRBD patients were conducted approximately every 12 months with all visits up to December 2024 included into the analysis unless patients were lost to follow-up or phenoconversion was determined earlier. Date of phenoconversion was set as timepoint, when patients fulfilled diagnostic criteria for PD, MSA or DLB^24,28^. Thus, time to phenoconversion was determined as period between baseline gait assessment and time of phenocoversion. Time of PD diagnosis or PSG proven RBD diagnosis until gait assessment (baseline) was determined as disease duration, respectively. All of the PD patients received dopaminergic treatment, while two of the iRBD patients received low levodopa doses due to restless legs syndrome.

### Gait analysis

Our walking protocol included the timed 25-foot walking (T25FW, 7.62 m distance) test for maximum walking speed and the 2-minute walk test to assess walking endurance (2-MWT)^29,30^ at self-selected walking speed over the length of the corridor of approximately 40 m. Furthermore, assisted testing of gait parameters was performed using the GAITRite pressure sensor walkway (CIR Systems Inc., CIR-Systems, Franklin, NJ, United States) with an active length of 793 cm, which has been validated previously^31^. Setting and setup was in line with the recommendation of the European GAITRite network group^32^. Weight, height and leg lengths for each leg was measured to calculate normalized gait parameters such as normalized gait velocity. Participants walked three times back and forth over the walkway with a turning point of approximately 2 m before and after the end of the walkway in a self-selected swift walking speed. All of these six gait trials were then analyzed and the average was computed as the overall outcome measures. To differentiate converters and non-converters only the first four trials were selected for better discriminative power. According to standardized protocols and recommendations, we evaluated a variety of temporal and spatio-temporal gait parameters in different domains including pace, variability, rhythm, postural control and asymmetry as suggested^9,11,22,33^ including gait velocity, step length, swing and stance as well as single and double support times^11,31,33–35^. The mean of both sides was calculated for several parameters such as stride lengths, step length, swing, stance, single and double support.

The gait cycle is defined as the complete forward movement from the initial contact of one foot as the stance and corresponding swing phase of the other foot until the terminal swing phase of the initial foot ends, thus the gait cycle time means the time between two contacts of the same foot in a forward motion gait. Velocity is defined as the distance covered over a certain time and is measured in meter / second (m/s), while normalized gait velocity is the velocity normalized to the mean length of both legs (LL/s). Single support is defined as the proportion of time of the gait cycle with contact of one foot only (% of gait cycle time), while swing is defined as the proportion of the gait cycle time the corresponding other foot is off the ground. Thus, double support means the proportion of the gait cycle time in which both feet are in contact with the ground simultaneously, while stance is defined as the proportion in which each separate foot is on the ground. Step length is measured as the distance between the heel of one and the other feet, while stride length is defined as the distance between two contacts of the same foot within the gait cycle^36^. Asymmetry of step length and stride length were calculated by the output system as discussed elsewhere^37^. Furthermore, we added another asymmetry measure computed as the difference between both sides/max (right, left)^38^.

### Statistics

Statistical analysis was performed using ANOVA with post-hoc Bonferroni-adjustment to compare all three groups for metric parameters, Kruskall-Wallis-test with post-hoc Mann-Whitney-U-test for non-parametric variables or χ^2^-/ Fisher’s exact test as appropriate. To compare baseline values of converters and non-converters for parametric variables unpaired students’-test and for non-parametric variables Mann-Whitney-U-test was performed. Correlations were calculated using Spearman correlation test. To identify relevant covariates of gait parameters in the iRBD cohort linear regression models were used, if appropriate. Furthermore, ROC-analysis was performed to analyze the sensitivity and specificity of gait parameters to determine probability of conversion. To examine the association between baseline gait parameters and time to conversion from gait assessment for the iRBD cohort, we used a Cox proportional hazards model. Relevant potential covariates including sex, age, height, BMI, UPDRS, ESS, disease duration as well as BDI-II were entered as covariates into this model. A stepwise selection process with significance levels for both entry and retention in the model set at 0.05 was used to select any significant predictors. However, none of these covariates showed a significant influence on the relevant parameters, thus we used a univariate Cox regression model. Baseline gait parameters were then added to the selected model. For continuous variables, risk of phenoconversion was then calculated with univariate Cox proportional hazards models to estimate hazard ratios (HRs) with 95% confidence intervals (95%CIs) and *p* values for pairwise comparisons. The proportional hazards assumption was tested using log-log plots. Medians of gait parameters were used to dichotomize the RBD cohort to analyze and illustrate differences in phenoconversion risks using log-rank-test and Kaplan-Meier curves.

Data were analyzed using the software program SPSS 29.0 or higher (SPSS Inc., Chicago, IL). If not mentioned otherwise, all results are presented as mean values ± standard deviation (SD) or numbers (n), the significance level was set at *P* < 0.05 (two-tailed test).

## Results

**Fig. 1:**
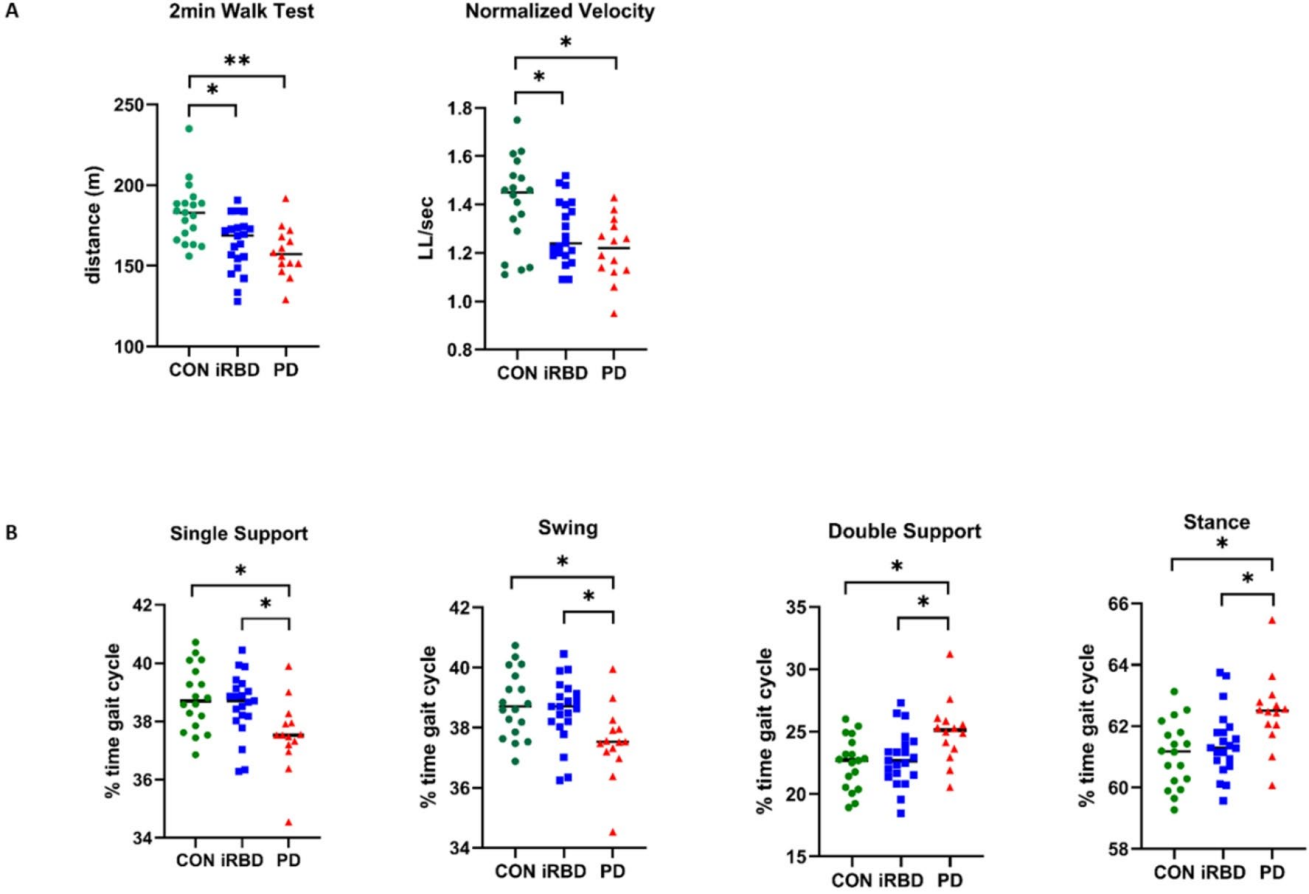
Comparison of gait parameters at baseline in all cohorts. (**A**) iRBD patients and PD patients showed a significantly lower walking endurance in the 2minutes Walk Test and gait velocity measured by the assisted gait analysis system (GAITrite) compared to healthy controls at baseline gait assessment. (**B**) Gait rhythm parameters as measured by the assisted gait analysis (GAITrite) showed significantly increased stance and double support times as well as decreased single support and swing time (as % of gait cycle time) in PD patients compared to the iRBD and control groups. For statistical analyses, ANOVAs with *post-hoc* t-test with Bonferoni-adjustment were performed. * represents *P* < 0.05. CON = Control group; iRBD = Clinically isolated REM Sleep Behavior Disorder group; m = meter; min = minutes; LL = leg length; PD = Parkinson’ disease group; sec = seconds; WT = Walk Test.

Patients and controls did not differ regarding basic demographic characteristics such as age and gender. As expected motor symptom load (UPDRS II and III) and levodopa equivalent dose (LED) but also daytime sleepiness (ESS) were significantly higher in PD compared to controls and iRBD patients, while features of RBD (RBDSQ) were more prominent in the iRBD cohort (Table 1). In iRBD nearly all gait parameters showed intermediate values compared to PD patients and controls. Thus, walking endurance as assessed by the 2 min walk test and mean normalized velocity as a parameter of gait pace measured by the GAITRite system were significantly lower in iRBD than in controls (Table 2; Fig. 1A). Gait rhythm parameters such as single support and double support as well as the respective swing and stance differed significantly between PD and iRBD patients as well as controls (Table 2; Fig. 1B). In PD further gait abnormalities compared to controls were detected including increased ambulation time and number of steps as well as decreased stride and step length (Table 2). However, none of the asymmetry measures showed significant differences between groups (Table 2).

**Table 1:** Baseline (time of 1st gait assessment) demographic and clinical characteristics of study cohorts.

	PD (*n* = 14)	iRBD (*n* = 21)	CON (*n* = 19)	*P* value
Demographics and clinical characteristics				
Age (years), mean ± SD (range)	70.3 ± 8.2 (57–82)	71.5 ± 6.6 (59–83)	68.5 ± 6.5 (58–78)	0.394^§^
Men/women, n (%)	9 (64%) / 5 (36%)	15 (71%) / 6 (29%)	10 (53%) / 9 (47%)	0.466^¥^
Height	1.73 ± 0.1	1.72 ± 0.1	1.71 ± 0.1	0.850^ϕ^
Weight	78.0 ± 12.8	77.5 ± 12.8	76.2 ± 16.2	0.925^§^
BMI	25.9 ± 3.1	26.0 ± 3.0	25.6 ± 3.5	0.790^ϕ^
Subjective symptom duration (years)	7.8 ± 5.7 (2–22)	4.8 ± 2.7 (1–13)	-	0.249^ϕϕ^
Disease duration (years since diagnosis, range)	**6.6 ± 5.8 (1–21)** ^††^	**1.4 ± 1.5 (0–5)** ^##^	-	**< 0.001** ^ϕϕ^
UPDRS total score	**29.6 ± 13.5** ^**††^	**5.1 ± 1.7** ^*##^	**2.7 ± 2.5**^†##^ **[13]**	**< 0.001** ^ϕ^
UPDRS part I	2.3 ± 2.1	2.1 ± 1.3^*^	1.2 ± 1.2^†^ [13]	0.111^ϕ^
UPDRS part II (ADL)	**8.1 ± 4.5** ^**††^	**0.8 ± 0.8** ^**##**^	**0.5 ± 0.8**^##^ **[13]**	**< 0.001** ^ϕ^
UPDRS part III (motor function)	**17.2 ± 9.3** ^**††^	**1.0 ± 1.0** ^**##**^	**0.3 ± 0.5**^**##**^ **[13]**	**< 0.001** ^ϕ^
UPDRS part IV	2.1 ± 2.7	1.1 ± 0.6	0.6 ± 0.8 [13]	0.094^ϕ^
Modified Hoehn & Yahr stage	**2.2 ± 0.4** ^**††^	**0** ^**##**^	**0** ^**##**^	**< 0.001** ^**¥**^
0	0	21	19	
1.5	1 (7%)			
2	9 (64%)			
2.5	2 (14%)			
3	2 (14%)			
MoCA	27.0 ± 2.8	27.4 ± 1.5	28.2 ± 0.9 [13]	0.284^ϕ^
RBDSQ	**3.4 ± 3.0**^†^ **[11]**	**6.8 ± 3.3** ^**#^	**1.7 ± 1.8**^††^ **[17]**	**< 0.001** ^ϕ^
BDI-II	5.5 ± 3.6 [12]	5.2 ± 4.7	3.5 ± 4.6 [15]	0.150^ϕ^
PSQI	4.9 ± 2.2 [12]	4.2 ± 2.4	4.1 ± 3.8 [17]	0.424^ϕ^
WHO-5 score	17.8 ± 4.5 [12]	17.5 ± 4.2 [20]	17.1 ± 5.4 [13]	0.995^ϕ^
PDSS-2	14.6 ± 8.5 [12]	9.4 ± 6.8 [8]	14.6 ± 9.1 [12]	0.344^ϕ^
ESS	**8.8 ± 4.7 [12]**	**5.1 ± 2.9** ^#^	7.4 ± 3.8	**0.023** **§**
PD-NMS	6.5 ± 5.1 [12]	5.1 ± 2.4 [20]	3.4 ± 3.0 [12]	0.064^ϕ^
SCOPA-AUT	10.6 ± 4.1 [14]	9.6 ± 4.9 [7]	7.7 ± 5.9 [12]	0.454
Dopaminergic medication	14 (100%)	2 (9%)	0	**< 0.001** ^**¥**^
Levodopa, n (%)	12 (86%)	1 (5%)	0	**< 0.001** ^¥^
Dopamine agonists, n (%)	8 (57%)	1 (5%)	0	**< 0.001** ^¥^
COMT inhibitors, n (%)	3 (21%)	0	0	**0.013** ^¥^
MAO-inhibitors, n (%)	8 (57%)	0	0	**< 0.001** ^¥^
Other, n (%)	2 (14%)	0	0	0.051^¥^
Baseline levodopa-equivalent dose (LED in mg per day), mean ± SD ^a^	678.7 ± 342.8**^††^ **(300–1335)**	**10.7 ± 39.2**^##^ **(0-175)**	**0 ± 0** ^##^	**< 0.001** ^ϕ^

Data are displayed as mean ± SD, range (min-max), numbers (n) or percentages (%). *P* values are from ^§^ANOVA, ^ϕ^Kruskall-Wallis-test, ^ϕϕ^Mann-Whitney U-test, ^¥^Chi-square test or ^¥¥^Fisher’s exact test.

Bold letters indicating significant P-values, ** represents *P* < 0.001, * *P* < 0.05 when compared to CON; ^##^ *P* ≤ 0.001, ^#^ *P* < 0.05 when compared to PD; ^††^ *P* ≤ 0.001, ^†^*P* < 0.05 when compared to iRBD (all from ANOVA with post-hoc Bonferroni analysis or Mann-Whitney U-test or Fisher’s exact test as appropriate). Numbers [n] are stated, if results were not available for all participants.

ADL=Activities of daily living; BMI = Body Mass Index; BDI=Beck’s Depression Inventory; CON=Controls; ESS=Epworth Sleepiness Scale; iRBD=clinical isolated REM Sleep Behavior Disorder; MoCA=Montreal Cognitive Assessment; PD=Parkinson’s disease; PDNMS=Parkinson’s Disease Non-Motor-Symptom Questionnaire; PSQI=Pittsburgh Sleep Quality Index; RBDSQ: REM Sleep Behavior Disorder Questionnaire; SD=Standard deviation; SDI = Sniffin sticks discrimination and identification score, WHO-5 = World Health Organization questionnaire; UPDRS=Unified Parkinson’s disease rating scale (part I: evaluation of mentation, behavior and mood; part II: activities of daily life; part III: motor function; part IV: complications).

^a^Levodopa equivalent dose was calculated according to Tomlinson and co-workers.

**Table 2:** Baseline gait metrics in all groups – for composite GAITRITE parameter of all trials (1–6).

	PD (*n* = 14)	iRBD (*n* = 21)	CON (*n* = 19)	*P* value
General gait parameter				
Timed 25-Foot Walk T1 (s)	5.0 ± 0.7	4.8 ± 0.9	4.4 ± 0.6	0.100^§^
Timed 25-Foot Walk T2 (s)	5.0 ± 0.9	4.9 ± 0.9	4.4 ± 0.6	0.110^§^
**2 min Walk Test (m)**	158.6 ± 15.5**	163.6 ± 17.1*	**182.5 ± 18.6** ^##†^	**< 0.001** ^**§**^
GAITRite parameter				
Distance (cm)	716.6 ± 24.4	714.7 ± 14.8	705.1 ± 17.4 [18]	0.109^ϕ^
**Ambulation time (s)**	6.6 ± 0.9*	6.1 ± 0.7	**5.6 ± 0.8**^**#**^[18]	**0.003** ^**§**^
**Velocity (cm/s)**	110.4 ± 12.2*	119.6 ± 13.8	**128.9 ± 15.6**^**#**^ [18]	**0.002** ^**§**^
**Mean Normalized Velocity (LL/s)**	1.2 ± 0.1*	1.3 ± 0.1*	**1.4 ± 0.2**^**#** †^ [18]	**0.002** ^**§**^
Cadence (steps/min)	110.5 ± 7.6	111.1 ± 7.4	116.8 ± 9.3 [18]	0.051^§^
**Number of steps**	12.1 ± 1.4*	11.2 ± 1.3	**10.8 ± 1.1**^**#**^ [18]	**0.049** ^ϕ^
Step length asymmetry (cm)	2.3 ± 1.2	2.6 ± 1.7	1.7 ± 1.0 [18]	0.083^ϕ^
Step time asymmetry (s)	0.02 ± 0.0	0.01 ± 0.0	0.01 ± 0.0 [18]	0.075^ϕ^
Cycle time asymmetry (s)	0.01 ± 0.0	0.01 ± 0.0	0.01 ± 0.0 [18]	0.168^ϕ^
Variation coefficient step length	1.9 ± 3.7	1.0 ± 4.7	0.5 ± 2.7 [18]	0.143
Variation coefficient stride length	0.4 ± 0.5	0.1 ± 0.5	0.2 ± 0.5 [18]	0.364
Combined data				
**Step length (cm)**	60.0 ± 5.7*	64.7 ± 7.3	**66.1 ± 5.4**^**#**^ [18]	**0.024** ^**§**^
Step time (s)	0.5 ± 0.0	0.5 ± 0.0	0.5 ± 0.0 [18]	0.086^§^
**Stride length (cm)**	120.4 ± 11.4*	129.9 ± 14.6	**132.8 ± 10.8**^**#**^ [18]	**0.023** ^**§**^
Gait cycle time (s)	1.1 ± 0.1	1.1 ± 0.1	1.0 ± 0.1 [18]	0.081^§^
**Swing time (%GCT)**	37.5 ± 1.2* ^†^	**38.6 ± 1.1** ^#^	**38.8 ± 1.1**^#^ [18]	**0.008** ^**§**^
**Stance time (%GCT)**	62.5 ± 1.2* ^†^	**61.4 ± 1.1** ^#^	**61.1 ± 1.1**^#^ [18]	**0.004** ^**§**^
Base width (cm)	9.9 ± 2.8	9.5 ± 2.3	8.7 ± 1.9 [18]	0.277^§^
**Single support time (%GCT)**	37.5 ± 1.2* ^†^	**38.6 ± 1.1** ^**#**^	**38.8 ± 1.1**^**#**^ [18]	**0.008** ^**§**^
**Double support time (%GCT)**	25.0 ± 2.5* ^†^	**22.8 ± 2.2** ^**#**^	**22.5 ± 2.1**^**#**^ [18]	**0.007** ^**§**^
**Step extremity ratio**	0.66 ± 0.05* ^†^	0.69 ± 0.06	**0.72 ± 0.05**^**#**^ [18]	**0.011** ^**§**^
Toe in toe out (degrees)	5.6 ± 4.0	5.6 ± 3.9	5.4 ± 4.3 [18]	0.902**ϕ**
Step function both sides	-1.7 ± 1.5	-1.1 ± 0.9	-1.4 ± 1.2 [18]	0.583**ϕ**
Diff Step Extremity Ratio	-0.3 ± 0.6	-0.8 ± 1.6	-0.1 ± 0.5 [18]	0.179ϕ

Combined data were calculated as the mean of GAITRITE parameter for both sides. Data are displayed as mean ± SD;

*P* values are from ^§^ANOVA with post-hoc Bonferroni adjustment or ϕKruskall-Wallis-test with post-hoc Mann-Whitney U-test. Bold letters indicating significant P-values, ** represents *P* < 0.001, * *P* < 0.05 when compared to CON; ^##^ *P* ≤ 0.001, ^#^ *P* < 0.05 when compared to PD; †*P* < 0.05 when compared to iRBD.

cm = centimeter, GCT = Gait Cycle Time, s = seconds, LL: Leg length; T1 = 1. Trial of 25 foot walk, T2 = second trial.

Correlation analyses showed associations of some of the gait parameters including distance and velocity with age in controls and PD, but not in the iRBD cohort (Supplementary Fig. 1A-C). Sex was associated with normalized velocity in the control group, while associated with number of step in the iRBD cohort. Furthermore, in all groups some associations of gait with autonomic symptoms (PDNMS) and non-motor symptoms were detected (Supplementary Fig. 1A-C). We did not find further associations of motor scores including UPDRS III as well as disease duration or cognitive function (MoCA) on gait parameters (Supplementary Fig. 1A-C) (MoCa), while Levodopa equivalent dose (LED) was associated with double support time in the PD cohort. Time to conversion from 1st gait assessment in the iRBD cohort was associated with a variety of gait parameters including single support, swing, distance, ambulation time, cycle time, double support and stance (Supplementary Fig. 1B).

**Fig. 2:**
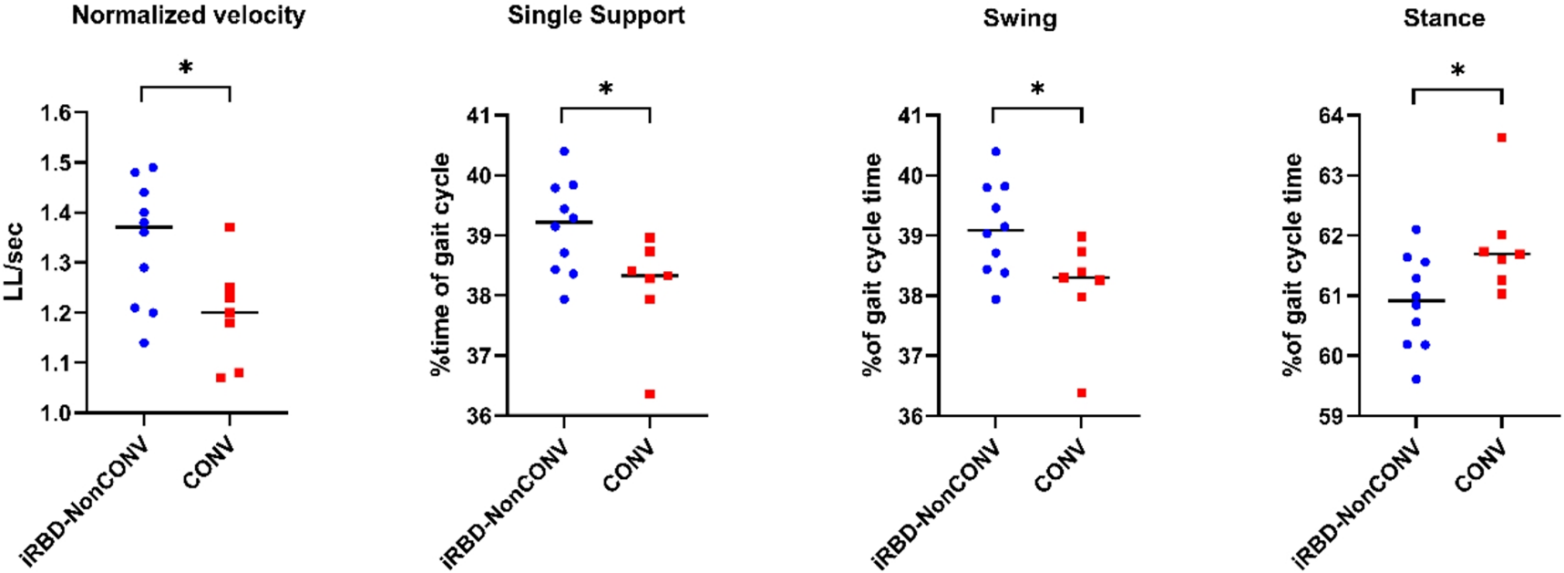
Gait parameters in iRBD converters (CONV) and non-converters (iRBD-NonCONV) at baseline. Baseline gait assessment showed significantly lower normalized gait velocity, decreased single support and swing time (% of gait cycle time) in RBD patients later converting to PD or DLB (classified as converters (CONV)) compared to non-converters (iRBD-NonCONV). Accordingly, stance (% of gait cycle time) was significantly elevated compared to non-converters. Statistical analysis was performed using Students’s t-tests. * represents *P* < 0.05. CON = Control group; iRBD = Clinically isolated REM Sleep Behavior Disorder group; m = meter; min = minutes; LL = leg length; PD = Parkinson’ disease group; sec = seconds; WT = Walk Test.

The mean overall follow-up period in iRBD patients was 6.0 ± 2.7 years (range 1–11) since polysomnographic diagnosis of RBD, whereas mean follow-up since gait analysis was 4.7 ± 2.2 years (Supplementary Table 1). After a mean follow-up of 3.7 ± 0.6 years (visit V3) after gait analysis 41% (*n* = 7) of all patients of which data were available for all visits (*n* = 17, excluding 4 patients lost to follow-up) phenoconverted to either PD (*n* = 4) or DLB (*n* = 3). At baseline gait assessment swing, stance and single support differed significantly between later converters and non-converters (Supplementary Table 2, Fig. 2). Of note, clinical parameters at baseline did not differ between converters and non-converters apart from Sniffin sticks identification values (Supplementary Table 3).

**Fig. 3:**
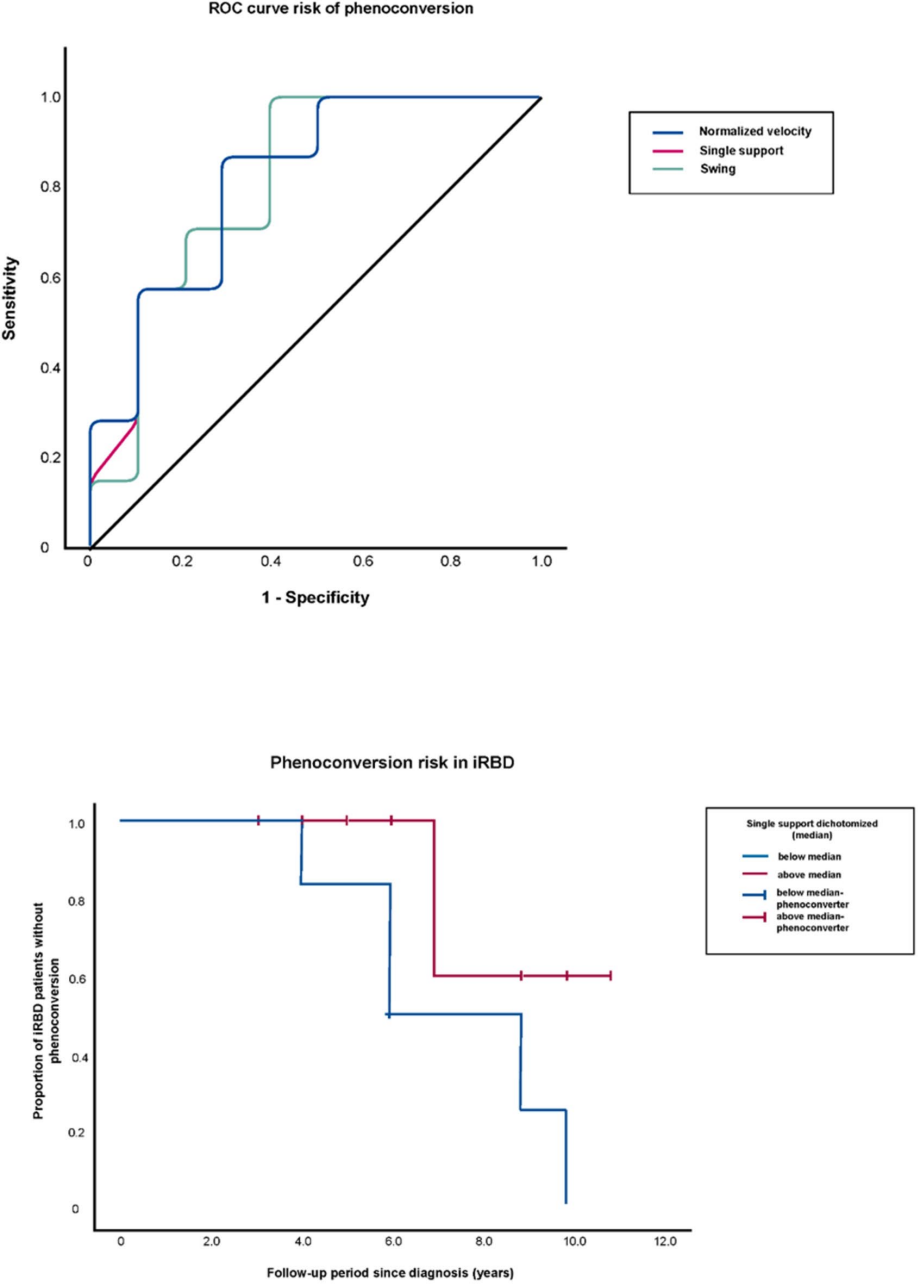
(**A**) ROC-analyses of gait parameters at baseline to predict likelihood of phenoconversion in the iRBD cohort. The figure shows the ROC analysis of gait parameters to predict phenoconversion. ROC analysis was performed with values of the first two gait trials amounting to the first 4 values of the gait analysis to better discriminate converters and non-converters. ROC analysis showed significant values for normalized velocity (AUC = 0.814, 95%CI [0.610–1.019], *P* = 0.003), single support (AUC = 0.821, 95%CI [0.620–1.023], *P* = 0.002) and swing (AUC = 0.814, 95%CI [0.607–1.022], *P* = 0.003) to predict phenoconversion. Furthermore, stance (AUC = 0.814, 95%CI [0.607–1.022], *P* = 0.003) and Step Extremity Ratio (AUC = 0.771, 95%CI [0.541–1.002], *P* = 0.021) showed significant ROC results (*data not shown*). (**B**) Kaplan Meier curve showing likelihood of phenoconversion within the iRBD cohort over time stratified by single support as the strongest predictor within all gait parameters. Log-rank test revealed the value of gait parameters to predict phenoconversion over time. Depicted here is the Kaplan Meier curve of the conversion risk over time in the iRBD group dichotomized into halves by the median cut-off for single support (*P* = 0.043).

ROC-analysis of normalized velocity (AUC = 0.814, 95%CI [0.610–1.019], *P* = 0.003), single support (AUC = 0.821, 95%CI [0.620–1.023], *P* = 0.002), swing (AUC = 0.814, 95%CI [0.607–1.022], *P* = 0.003) and stance (AUC = 0.814, 95%CI [0.607–1.022], *P* = 0.003) showed excellent sensitivity and specificity to identify converters (Fig. 3A). In univariate Cox regression analysis, single support demonstrated a significant potential to identify converters with a significantly diminished hazard ratio for the risk of phenoconversion within 3.7 ± 0.6 years of follow-up underlining the inversely proportional relationship between single support (% of gait cycle time) and conversion risk (HR 0.103, [95%CI 0.015–0.690], *P* = 0.019) (Supplementary Table 4). In accordance, swing (HR 0.104 [95%CI 0.015–0.719], *P* = 0.022) indicated a decreased risk of phenoconversion, while consequently stance was associated with an increased conversion risk (HR 9.323 [95%CI 1.394–62.362], *P* = 0.021). Furthermore, Cox regression analysis also showed significant results for single support to identify phenoconverters even at later timepoints (up to 6.3 ± 1.3 years past gait analysis (HR 0.320 [95%CI 0.104– 0.980], *P* = 0.046, Supplementary Table 4). Log-rank tests of the dichotomized RBD cohort using the median cut-offs showed significant elevated phenoconversion risk in the iRBD subcohort with lower scores of single support times compared to patients with higher single support times (Fig. 3B).

## Discussion

Our study used gait assessment and longitudinal clinical follow-up data to determine the potential of a single assessment of gait to predict progression and phenoconversion in iRBD patients. The iRBD cohort showed significantly impaired normalized gait velocity and walking endurance in comparison to the control group. This is in contrast to the clinical scales such as UPDRS III, which demonstrated only minimal motor abnormalities in iRBD patients without significant differences to the control group. While step and stride length showed significant abnormalities only in PD but not iRBD compared to controls, we were able to detect differences in gait rhythm parameters such as swing, single support and stance in our PD cohort compared to controls and iRBD. This is in contrast to earlier studies, which only showed differences in double support times^18,39^. Our PD cohort comprised of patients with mild to moderate disease stages (H&Y I-III) and a moderate disease duration of around 6.5 years. Thus, our results are in line with previously published data demonstrating early gait disturbances with progression over time^13,14,16,21,40^. Interestingly, all of these gait disturbances were already unmasked at normal walking speed and not only on dual-task walking^22,38^, emphasizing the sensitivity of gait assessment to detect minimal abnormalities even in early neurodegenerative stages^41–43^ particularly based on decreased velocity, stride and step length in early^20,21,40,44–46^ and *de novo* stages of PD^47,48^.

However, data on gait disturbances in iRBD patients are less numerous and conclusive. In line with our results, reduced gait velocity has been demonstrated by clinical tests such as the “Timed Up and Go” (TUG)^3^ or assisted gait evaluation systems^37,41^ in iRBD. However, at least one of these studies included only probable RBD patients as suspected by questionnaires^37^, thus results are not as valid as our study. In contrast to our results, a single-center cross-sectional study by Ehgoetz Martens and colleagues found no differences in gait velocity in iRBD patients and healthy controls in regular walking conditions^22^. These differences might be due to different assessment technologies and low participant numbers in both trials. However, decreased velocity seems to be a non-specific feature of overall declining health associated with increased morbidity, mortality and dementia indicating brainstem and cholinergic dysfunction^9,49–51^. Since our raw data of velocity lie within ranges of previously published reference values for PD patients as well as healthy elderly a bias towards more severely affected cohorts can be ruled out^11,34,52^.

While some groups reported increased gait variability parameters in iRBD patients compared to controls^22^, we as well as others were not able to confirm these results^53^. This might be due to methodological differences, since we did not include dual-task and fast-walking conditions in our study design, which seem to unmask gait variability measures. On the contrary, gait rhythm parameters such as swing and single support differed significantly between PD compared to iRBD and controls, but not between iRBD and controls. This is in contrast to Del Din and colleagues, who reported significant alterations of gait asymmetry and gait rhythm parameters in iRBD compared to controls^41^. While the use of different gait assessment tools could be partly responsible for these inconsistencies, more importantly our study also included female iRBD patients and excluded all RBD participants with more advanced motor abnormalities, while Del Din and colleagues assessed RBD patients with an average MDS-UPDRS III score of 6 ± 6.7 points.

The most relevant finding of the present study is that iRBD patients, which phenoconverted within the next 3.7 ± 0.6 years, were distinguishable from non-converters based on normalized gait velocity and gait rhythm parameters such as single support and swing. ROC analysis of these gait rhythm parameters showed a high sensitivity and specificity to identify patients at risk of conversion. Furthermore, COX regression analysis underlined the predictive value of single support to identify patients at risk of phenoconversion.

To our knowledge, the only other study determining longitudinal outcome based on assisted gait evaluation in iRBD did not detect any significant differences between the iRBD cohort and controls at baseline. Only after dividing the iRBD group, later phenoconverters differed significantly from the control cohort regarding gait variability parameters such as stride length and stride velocity variability, but not in any other gait domains including pace and rhythm, which is in contrast to our findings^43^. However, in line with our results, another study showed that gait abnormalities were identifiable approximately four years prior to diagnosis of PD in a cohort of community-dwelling elderly^54^. Earlier studies on progression in iRBD also showed that slight motor abnormalities occur within a time period of 4.5 years prior to diagnosis of a manifest α-synucleinopathy^4,55^. The value of monitoring motor progression by assisted gait assessment is further underlined by other recent studies predicting motor progression in PD^13,56^ based on gait rhythm parameters such as swing and double support^13^. Thus, an insidious transition from mild motor abnormalities and subtle gait disturbances in iRBD to a manifest α-synucleinopathy, particularly PD, with progressive worsening of motor and gait function can be postulated.

Limitations of our study are the exploratory character with only small participant numbers in a monocentric trial design, which limit the generalizability of our results. Furthermore, we lost a significant number of patients to follow-up, which is a shortcoming in many longitudinal trials. The use of a floor based pressure walkway is also a limitation as this method allows laboratory based standardized assessments, but no long-term assessments at home reflecting real world conditions. Further limitations include the lack of longitudinal gait assessment and the low patient numbers limiting the potential of gait parameters to evaluate specific patterns in different α-synucleinopathy phenoconverters.

In summary, although we were not able to detect significant differences between healthy controls and iRBD patients apart from normalized gait velocity and gait endurance, we could show an intermediate profile of iRBD patients regarding all relevant spatio-temporal gait parameters and particularly gait rhythm parameters. Our findings are in line with other studies, which were also not able to detect differences in gait between normal controls and iRBD at regular single-task walking conditions^22,42^. Studies in which further differences in spatio-temporal parameters between iRBD and controls were detected either used different gait protocols such as dual-task walking conditions or far more advanced disease stages of RBD were included^38,41^. Furthermore, we were able to predict phenoconversion based on baseline gait assessment. Thus, our results underline the potential of assisted gait evaluation to detect and quantify abnormalities in iRBD patients at an early disease stage, to monitor motor progression over time and to predict phenoconversion, which could be useful for patient stratification in clinical studies investigating disease-modifying drugs and thus should be further evaluated in large, multicenter trials.

## Supplementary Information

Below is the link to the electronic supplementary material.

Supplementary Material 1

## Data Availability

All data generated or analyzed during this study are included in this published article and its supplementary information files.
