# Supplementary material for: Gait dysfunction as an early marker of phenoconversion in REM sleep behavior disorder
Source: Sci Rep. 2026 Feb 9;16:5614. doi: 10.1038/s41598-026-37925-w (PMC12891670; doi:10.1038/s41598-026-37925-w)
Supplement: Supplementary file 1 — Supplementary Material 1 [file 41598_2026_37925_MOESM1_ESM.docx]

**SUPPLEMENTARY FILES**

**SUPPLEMENTARY TABLES**

**Supplementary Table 1: Demographics and clinical characteristics of the iRBD cohort at time of 1^st^ gait assessment** (**baseline) and data at baseline for later converters and non-converters.**

| **Baseline parameters** | **iRBD (n=17)** | **iRBD at V3 (n=10)** | **Converters at V3 (n=7)** | ***P* values** |
| --- | --- | --- | --- | --- |
| **Demographics** |  |  |  |  |
| Age (years), mean±SD (range) | 71.8±6.2 (63-83) | 70.5±7.0 (63-83) | 73.7±4.7 (65-78) | 0.624ᶲ |
| Men/women, n (%) | 14 (82%) / 3 (18%) | 7 (70%) / 3 (30%) | 7 (100%) / 0 (0%) | 0.228^¥¥^ |
| Reported symptom duration (years) | 4.6±2.9 (1-13) | 4.7±1.6 | 4.5±4.3 | 0.417ᶲ |
| Disease duration (since diagnosis) (years) | 1.3±1.6 (0-5) | 1.5±2.0 | 1.0±0.8 | 0.887ᶲ |
| Overall follow-up period since diagnosis | 6.6±2.5 (3 – 11) | 6.3±2.8 | 7.0±2.0 | 0.417ᶲ |
| Overall follow-up period since baseline | 5.4±1.9 (2 - 8) | 4.8±1.9 | 6.1±1.7 | 0.161ᶲ |
| Time period between reported symptom onset and V3 | 8.7±3.0 (4 – 16) [15] | 8.5±2.0 [8] | 9.0±4.0 | 0.779ᶲ |
| Time period between diagnosis and V3 | 5.1±1.8 (3 – 9) [15] | 5.5±2.3 [8] | 4.6±1.0 | 0.694ᶲ |
| Time period between BL (1^st^ gait  assessment) and V3 | 3.7±0.6 (3 – 5) [15] | 3.8±0.5[8] | 3.7±0.8 | 0.867ᶲ |
| Time period to conversion since reported symptom onset |  |  | 9.6±3.7 (4 - 16) |  |
| Time period to conversion since diagnosis |  |  | 5.1±1.3 (3 – 7) |  |
| Time period to conversion since baseline |  |  | 4.6±1.4 (3 – 7) |  |
| Levodopa-equivalent dose | 13.2±43.4 (0-175) | 17.5±55.3 | 7.1±18.9 | 0.962ᶲ |
| **Clinical characteristics at baseline** |  |  |  |  |
| UPDRS total score | 5.3±1.7 | 4.9±1.6 | 5.9±1.8 | 0.364ᶲ |
| UPDRS part I | 2.4±1.3 | 2.2±1.3 | 2.7±1.3 | 0.536ᶲ |
| UPDRS part II (ADL) | 0.8±0.7 | 0.6±0.7 | 1.1±0.7 | 0.161ᶲ |
| UPDRS part III (motor function) | 1.0±1.1 | 0.9±0.9 | 1.1±1.5 | 0.962ᶲ |
| UPDRS part IV | 1.1±0.6 | 1.2±0.4 | 0.9±0.7 | 0.364ᶲ |
| MoCa | 27.2±1.4 | 27.6±1.0 | 26.7±1.8 | 0.230ᶲ |
| RBDSQ | 6.4±3.3 | 6.6±3.4 | 6.0±3.4 | 0.887ᶲ |
| BDI-II | 5.2±5.2 | 3.9±5.4 | 7.6±4.5 | 0.109ᶲ |
| PSQI | 3.9±2.4 | 4.3±3.1 | 3.4±1.1 | 0.813ᶲ |
| WHO-5 score | 17.3±4.6 | 17.8±4.2 | 16.5±5.5 [6] | 0.562ᶲ |
| ESS | 5.4±3.1 | 4.5±3.4 | 6.7±2.1 | 0.161ᶲ |
| PD-NMS | 5.0±2.3 | 5.3±2.3 | 4.6±2.5 | 0.475ᶲ |
| **Sniffin sticks identification** | **6.9±3.6 [16]** | **8.6±3.5 [9]** | **4.9±2.8** | **0.031ᶲ** |
| **Sniffin sticks SDI** | **19.6±8.2 [15]** | **22.8±8.5 [9]** | **14.8±5.1 [6]** | **0.050ᶲ** |

*P* values are from ᶲᶲMann-Whitney U-test, ^¥^Chi-square test or ^¥¥^Fisher’s exact test comparing converter and non-converter as appropriate. Missing patients at follow-up (n=4) are lost-to-follow-up.

Bold letters indicating significant P-values, * represents *P*<0.05 comparing iRBD non-converters and converters.

**Abbreviations:** ADL=Activities of daily living; BDI=Beck’s Depression Inventory; CON=Controls; ESS=Epworth Sleepiness Scale; iRBD=clinical isolated REM Sleep Behavior Disorder; MoCA=Montreal Cognitive Assessment; PD=Parkinson’s disease; PDNMS=Parkinson’s Disease Non-Motor-Symptom Questionnaire; PSQI=Pittsburgh Sleep Quality Index; RBDSQ: REM Sleep Behavior Disorder Questionnaire; SD=Standard deviation; SDI = Sniffin sticks discrimination and identification score, WHO-5=World Health Organization questionnaire; UPDRS=Unified Parkinson’s disease rating scale (part I: evaluation of mentation, behavior and mood; part II: activities of daily life; part III: motor function; part IV: complications); V3 = follow-up visit V3 with a mean time difference of 3.6 ± 0.7years from baseline (1st gait assessment).

**Supplementary Table 2: Gait metrics of trials 1-4 at baseline for all iRBD patients at baseline for the overall cohort and differentiating (between) later converters and non-converters (at follow up Visit (V3))**

|  | **iRBD (n=17)** | | **Non-Converter (n=10)** | | **Converter**  **(n=7)** | | ***P* values** | |
| --- | --- | --- | --- | --- | --- | --- | --- | --- |
| **General gait parameter** | |  | |  | |  | |  |
| Timed 25-Foot Walk T1 (s) | 4.8±1.0 | | 4.8±1.2 | | 4.8±0.5 | | 0.163ᶲ | |
| Timed 25-Foot Walk T2 (s) | 4.9±0.9 | | 4.8±1.1 | | 4.9±0.4 | | 0.412ᶲ | |
| 2 Minute Walk Test (cm) | 164.8±17.0 | | 163.3±20.8 | | 167.0±10.6 | | 0.785ᶲ | |
| **GAITRITE parameter** |  | |  | |  | |  | |
| Distance (m) | 711.2±18.2 | | 712.4±11.7 | | 709.5±26.0 | | 0.962ᶲ | |
| Ambulation time (s) | 6.0±0.7 | | 5.8±0.7 | | 6.3±0.8 | | 0.217^§^ | |
| Velocity (cm/s) | 119.8±13.6 | | 123.9±14.5 | | 114.0±10.7 | | 0.147^§^ | |
| **Mean Normalized Velocity (LL/s)** | 1.3±0.1 | | **1.3±0.1*** | | **1.2±0.1*** | | **0.027^§^** | |
| Cadence (steps/min) | 109.9±7.2 | | 111.3±6.5 | | 108.1±8.4 | | 0.388^§^ | |
| Number of steps | 11.0±1.3 | | 10.8±1.3 | | 11.3±1.2 | | 0.270ᶲ | |
| Step length asymmetry (cm) | 2.8±1.8 | | 2.4±1.4 | | 3.3±2.2 | | 0.536ᶲ | |
| Step time asymmetry (s) | 0.02±0.0 | | 0.02±0.0 | | 0.02±0.0 | | 0.417ᶲ | |
| Cycle time asymmetry(s) | 0.01±0.0 | | 0.02±0.0 | | 0.01±0.0 | | 0.887ᶲ | |
| **Combined data** |  | |  | |  | |  | |
| Step length (cm) | 65.5±7.4 | | 66.9±7.9 | | 63.5±6.6 | | 0.370^§^ | |
| Step time (s) | 0.5±0.0 | | 0.5±0.0 | | 0.6±0.0 | | 0.319^§^ | |
| Stride length (cm) | 131.5±14.8 | | 134.3±15.8 | | 127.5±13.3 | | 0.368^§^ | |
| Gait cycle time (s) | 1.1±0.1 | | 1.1±0.1 | | 1.1±0.1 | | 0.308^§^ | |
| **Swing time (%GCT)** | 38.7±0.9 | | **39.1±0.8*** | | **38.1±0.8*** | | **0.027**^§^ | |
| **Stance time (%GCT)** | 61.3±0.9 | | **60.9±0.8*** | | **61.8±0.8*** | | **0.029**^§^ | |
| Base width (cm) | 9.2±2.3 | | 8.7±2.9 | | 10.0±0.8 | | 0.273ᶲ | |
| **Single support time (%GCT)** | 38.7±0.9 | | **39.1±0.8*** | | **38.1±0.9*** | | **0.024**^§^ | |
| Double support time (%GCT) | 22.5±1.9 | | 21.9±2.0 | | 23.5±1.5 | | 0.103^§^ | |
| Step extremity ratio | 0.7±0.1 | | 0.7±0.1 | | 0.7±0.0 | | 0.055^§^ | |
| Toe in toe out (degrees) | 5.4±4.3 | | 4.2±4.0 | | 7.2±4.4 | | 0.270ᶲ | |
| Step function both sides | -1.0±1.0 | | -0.7±0.8 | | -1.5±1.1 | | 0.088ᶲ | |
| Diff Step Extremity Ratio | -1.0±1.6 | | -0.6±0.9 | | -1.6±2.2 | | 0.669ᶲ | |

Data are displayed as mean±SD*, P* values are from ^§^student’s t-test, ᶲMann-Whitney U-test, ^¥^Chi-square test or ^¥¥^Fisher’s exact test comparing converter and non-converter

Bold letters indicating significant P-values, * represents *P*<0.05 comparing iRBD non-converters and converters.

**Abbreviations:** cm = centimeter; GCT = Gait Cycle Time; s = seconds; T1 = 1. Trial of 25 foot walk; T2 = second trial

|  | **Parameter at baseline** | | | | **Parameter at V3** | | | |
| --- | --- | --- | --- | --- | --- | --- | --- | --- |
|  | **Total RBD cohort (n=17)** | **iRBD at V3 (n=10)** | **Converter at V3 (n=7)** | ***P* value** | **Total RBD cohort [17]** | **iRBD (n=10)** | **Converter (n=7)** | ***P* value** |
| **Clinical characteristics** |  |  |  |  |  |  |  |  |
| UPDRS total score | 5.3±1.7 | 4.9±1.6 | 5.9±1.8 | 0.364ᶲ | 8.8±6.0 [13] | **5.4±2.4 [7]** | **12.7±6.8 [6]** | **0.035ᶲ** |
| UPDRS part I | 2.4±1.3 | 2.2±1.3 | 2.7±1.3 | 0.536ᶲ | 2.7±1.5 [13] | 2.7±1.6 | 2.7±1.5 | 0.945**ᶲ** |
| UPDRS part II (ADL) | 0.8±0.7 | 0.6±0.7 | 1.1±0.7 | 0.161ᶲ | 0.7±1.0[13] | 0.4±0.8 | 1.0±1.3 | 0.445**ᶲ** |
| UPDRS part III (motor function) | 1.0±1.1 | 0.9±0.9 | 1.1±1.5 | 0.962ᶲ | 4.0±5.1 [13] | **0.9±0.9** | **7.7±5.5** | **0.022ᶲ*** |
| UPDRS part IV | 1.1±0.6 | 1.2±0.4 | 0.9±0.7 | 0.364ᶲ | 1.4±0.7 [13] | 1.3±0.8 | 1.4±0.5 | 0.628**ᶲ** |
| MoCA | 27.2±1.4 | 27.6±1.0 | 26.7±1.8 | 0.230ᶲ | 27.0±2.5 [13] | 27.9±2.3 | 26.0±2.5 | 0.138**ᶲ** |
| RBDSQ | 6.4±3.3 | 6.6±3.4 | 6.0±3.4 | 0.887ᶲ | 6.2±3.0 [10] | 6.2±1.9 [6] | 6.3±4.6 [4] | 0.914**ᶲ** |
| BDI-II | 5.2±5.2 | 3.9±5.4 | 7.6±4.5 | 0.109ᶲ | 5.9±5.8 [7] | 7.0±7.7 [4] | 4.3±2.5 [3] | 0.857**ᶲ** |
| PSQI | 3.9±2.4 | 4.3±3.1 | 3.4±1.1 | 0.813ᶲ | 4.5±1.7 [10] | 4.2±2.1[6] | 5.0±0.8 [4] | 0.257**ᶲ** |
| WHO-5 score | 17.3±4.6 | 17.8±4.2 | 16.5±5.5 [6] | 0.562ᶲ | 16.0±5.0 [10] | 14.5±6.0 [6] | 18.3±1.7 [4] | 0.476**ᶲ** |
| ESS | 5.4±3.1 | 4.5±3.4 | 6.7±2.1 | 0.161ᶲ | 3.9±3.5 [10] | 4.3±4.5 [6] | 3.3±1.5 [4] | 0.914**ᶲ** |
| PD-NMS | 5.0±2.3 | 5.3±2.3 | 4.6±2.5 | 0.475ᶲ | 5.6±3.3 [9] | 6.6±3.0 [5] | 4.3±3.5 [4] | 0.286**ᶲ** |
|  |  |  |  |  |  |  |  |  |

**Supplementary Table 3:** **Demographics and clinical characteristics of the whole iRBD cohort at time of 1^st^ gait assessment** (**baseline) as well as characteristics for later converters and non-converters (at follow-up visit 3 (V3)) shown separately. Missing data belong to patients lost-to-follow-up (n=4)**

Data are displayed as mean ± SD. *P* values are from ᶲMann-Whitney U-test comparing converter and non-converter at baseline and follow-up visit V3. Missing patients at follow-up (n=4) are lost-to-follow-up. Bold letters indicating significant P-values, * represents *P*<0.05 comparing iRBD non-converters and converters.

**Abbreviations:** ADL=Activities of daily living; BDI=Beck’s Depression Inventory; ESS=Epworth Sleepiness Scale; iRBD=clinical isolated REM Sleep Behavior Disorder; MoCA=Montreal Cognitive Assessment; PD-NMS=Parkinson’s Disease Non-Motor-Symptom Questionnaire; PSQI=Pittsburgh Sleep Quality Index; SD=Standard deviation; WHO-5=World Health Organization questionnaire; UPDRS=Unified Parkinson’s disease rating scale (part I: evaluation of mentation, behavior and mood; part II: activities of daily life; part III: motor function; part IV: complications); V3 = follow-up visit V3 with a mean time difference of 3.6 ± 0.7years from baseline (1^st^ gait assessment)

**Supplementary Table 4:** **Data of Cox regression analysis of gait parameter in a stepwise approach for the iRBD cohort predicting risk of phenoconversion**

| **Gait parameter continuous variables** |  |  |
| --- | --- | --- |
|  | **follow-up since diagnosis** |  |
|  | **HR [95% CI]** | ***P* values** |
| **Visit 3 (after a mean follow-up of 3.7±0.6 years)** |  |  |
| **Normalized velocity** | 0.002 [0.000 – 4.517] | 0.114 |
| **Single support** | 0.103 [0.015 – 0.690] | **0.019 *** |
| **Double support** | 1.451 [0.880 – 2.392] | 0.144 |
| **Swing** | 0.104 [0.015 – 0.719] | **0.022 *** |
| **Stance** | 9.323 [1.394 – 62.362] | **0.021 *** |
| **Step extremity Ratio** | 0.000 [0.000 – 45.708] | 0.095 |
| **Visit 4 (mean follow-up of 5.3±0.8years)** |  |  |
| **Single support** | 0.338 [0.116 – 0.980] | **0.046*** |
| **Double support, Swing, Stance** |  | n.s. |
| **Visit 5 (mean follow-up of 6.3±1.3years)** |  |  |
| **Single support** | 0.320 [0.104 – 0.980] | **0.046*** |
| **Double support, Swing, Stance** |  | n.s. |

Data are shown for univariate Cox regression analysis using a stepwise approach to predict phenoconversion risk. Gait variables of the first two trials amounting to four single measures were used to calculate the HR with 95% confidence interval to predict phenoconversion at visit V3, V4 and V5.

Bold letters indicating significant P-values, * represents *P*<0.05

**SUPPLEMENTARY FIGURES**

**Supplementary Figure 1:**

**
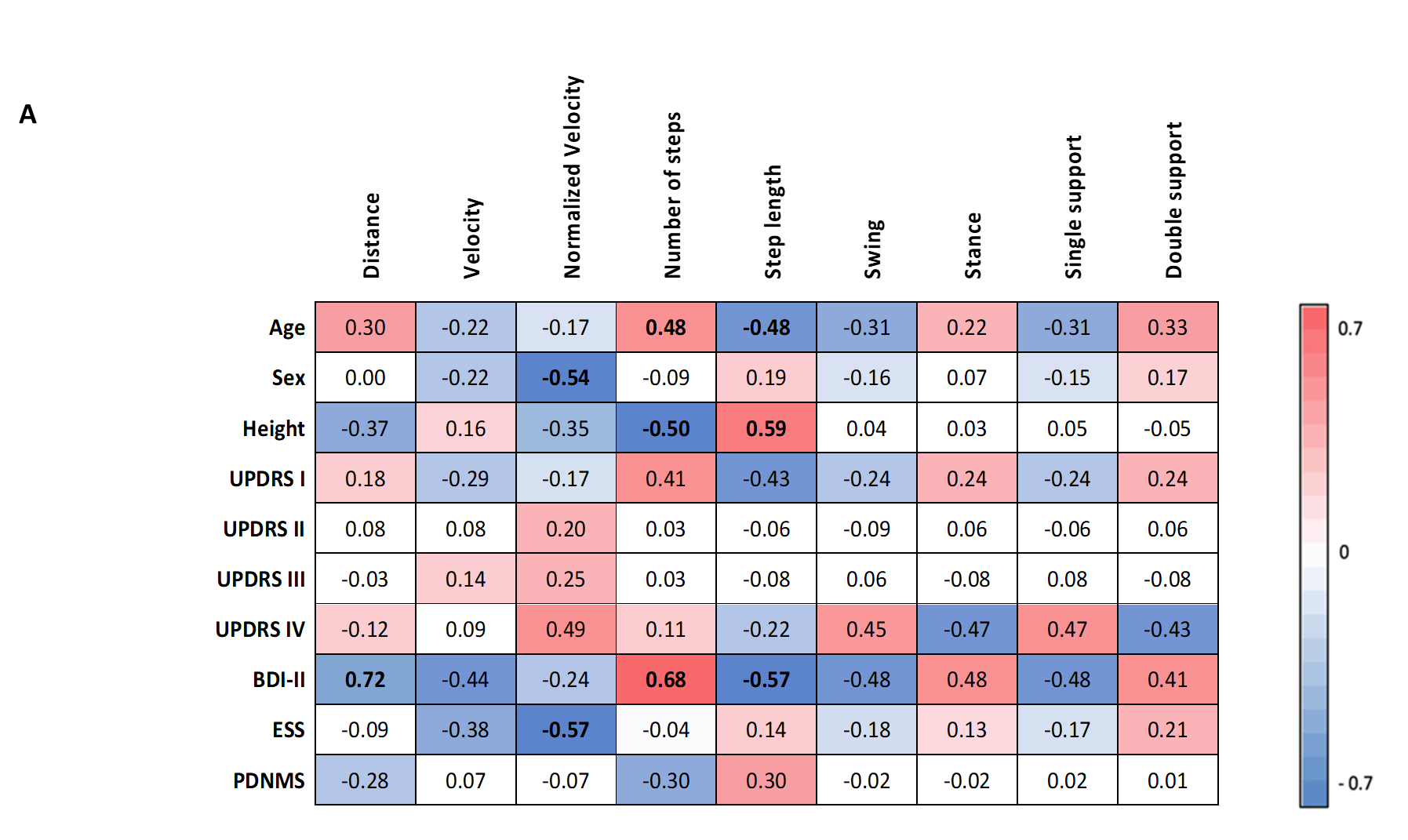
**

**Supplementary Figure 1A: Correlations of selected gait parameters with clinical and demographic data within the control cohort**

Correlations are based on Spearmans -coefficient correlation showing the correlations of selected clinical and demographic data with gait parameters.

ρ= Spearmans rho. Significant values are marked in bold (*P*<0.05, two sided).

**Abbreviations:** BDI=Beck’s Depression Inventory; CON=Controls; ESS=Epworth Sleepiness Scale; PDNMS=Parkinson’s Disease Non-Motor-Symptom Questionnaire; UPDRS=Unified Parkinson’s disease rating scale (part I: evaluation of mentation, behavior and mood; part II: activities of daily life; part III: motor function; part IV: complications)

**
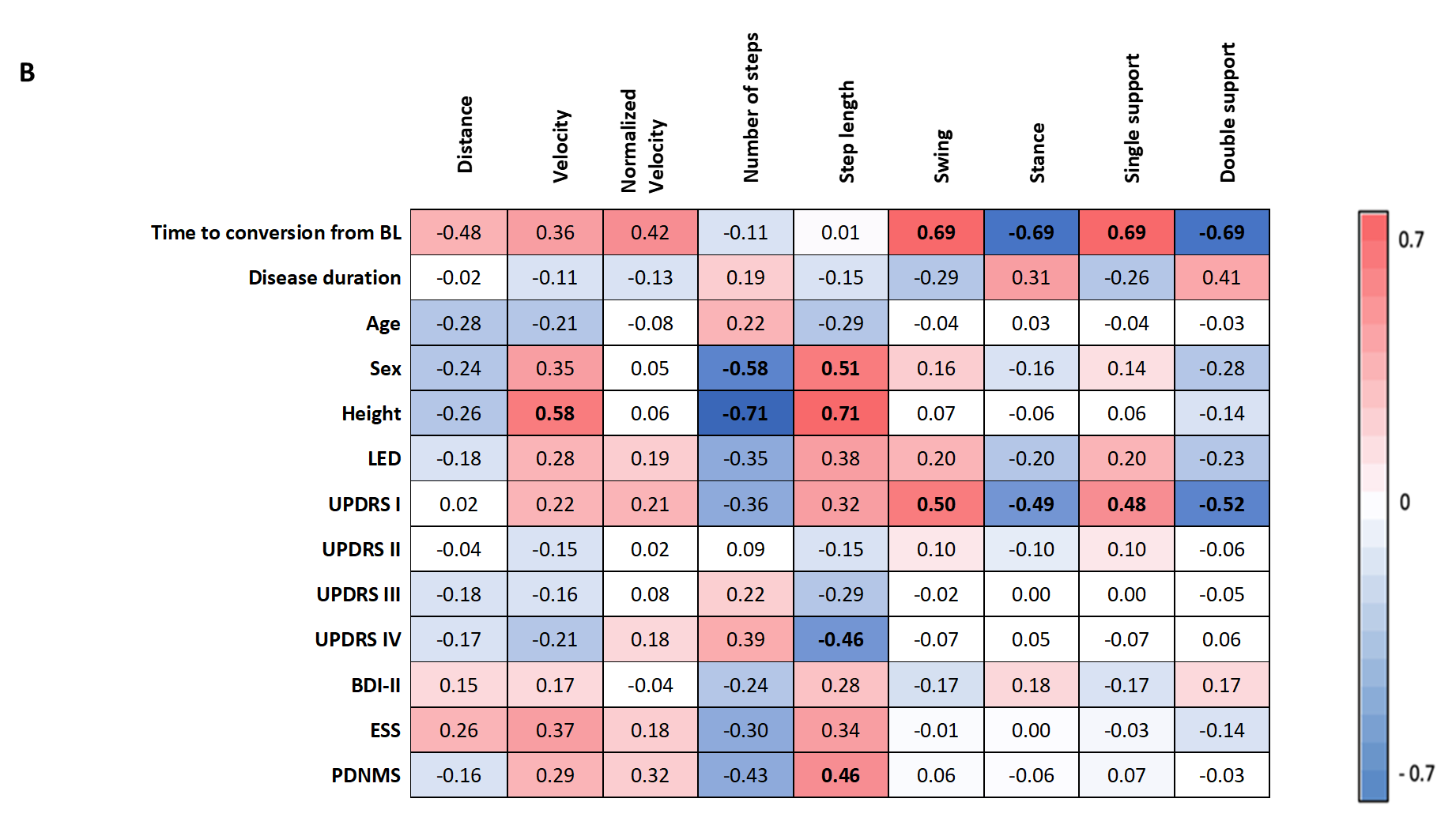
**

**Supplementary Figure 1B: Correlations between selected gait features and demographic and clinical data for the iRBD cohort**

Significant values are marked in bold (p<0.05, two sided). Correlations are based on Spearmans -coefficient correlation.

ρ= Spearmans rho. Significant values are marked in bold (*P*<0.05, two sided).

**Abbreviations:** BDI=Beck’s Depression Inventory; ESS=Epworth Sleepiness Scale; iRBD=clinical isolated REM Sleep Behavior Disorder; PDNMS=Parkinson’s Disease Non-Motor-Symptom Questionnaire; UPDRS=Unified Parkinson’s disease rating scale (part I: evaluation of mentation, behavior and mood; part II: activities of daily life; part III: motor function; part IV: complications)


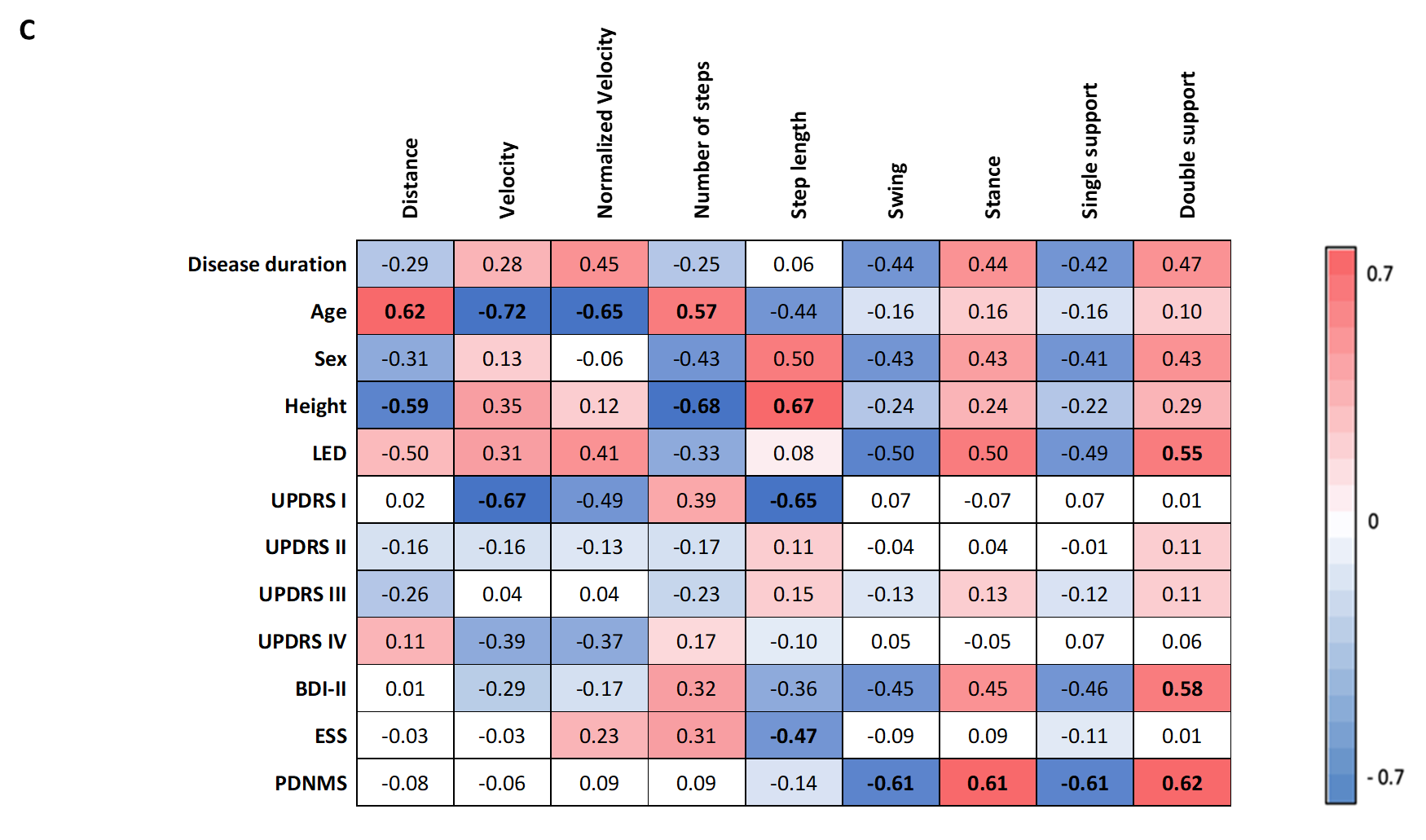


**Supplementary Figure 1C: Correlations between selected gait features and demographic and clinical data for the PD cohort**

Significant values are marked in bold (p<0.05, two sided). Correlations are based on Spearmans -coefficient correlation. ρ= Spearmans rho. Significant values are marked in bold (*P*<0.05, two sided).

**Abbreviations:** BDI=Beck’s Depression Inventory; ESS=Epworth Sleepiness Scale; PD=Parkinson’s disease, PDNMS=Parkinson’s Disease Non-Motor-Symptom Questionnaire; UPDRS=Unified Parkinson’s disease rating scale (part I: evaluation of mentation, behavior and mood; part II: activities of daily life; part III: motor function; part IV: complications)
